# In an interconnected world: joint research priorities for the environment, agriculture and infectious disease

**DOI:** 10.1186/2049-9957-3-2

**Published:** 2014-01-28

**Authors:** Bianca Brijnath, Colin D Butler, Anthony J McMichael

**Affiliations:** 1 Department of General Practice, School of Primary Health Care, Monash University, Building 1, 270 Ferntree Gully Road, Notting Hill, Victoria 3168, Australia; 2 Faculty of Health, University of Canberra, Canberra, Australia; 3 National Centre for Epidemiology and Population Health, Australian National University, Canberra, Australia

**Keywords:** Agriculture, Environment, Infectious disease, Research priorities, TDR

## Abstract

In 2008 the UNICEF/UNDP/World Bank/WHO Special Programme for Research and Training in Tropical Diseases (TDR) commissioned ten think-tanks to work on disease-specific and thematic reference groups to identify top research priorities that would advance the research agenda on infectious diseases of poverty, thus contributing to improvements in human health. The first of the thematic reference group reports – on environment, agriculture and infectious diseases of poverty – was recently released. In this article we review, from an insider perspective, the strengths and weaknesses of this thematic reference group report and highlight key messages for policy-makers, funders and researchers.

## Multilingual abstract

Please see Additional file 1 for translations of the abstract into the six official working languages of the United Nations.

## Background

In early 2013, the UNICEF/UNDP/World Bank/World Health Organization Special Programme for Research and Training in Tropical Diseases (TDR) released a technical report on research priorities for the environment, agriculture and infectious diseases of poverty. This report is one of a paired series of thematic reports and disease-specific reports commissioned by TDR, intended to foster an effective and more coordinated global research effort on infectious diseases of poverty that will lead to better health.

To date, disease-specific reports on topics such as Chagas disease, tuberculosis, helminth infections and zoonoses have been released. This report is the first from the *thematic* series to be published. That series sought, particularly, to understand the inter-linkages, often interactions, between different influences on infectious diseases of poverty. This report is the culmination of over four years of work by more than twelve leading researchers in environment, agriculture and infectious disease. Through their expertise material from disciplines including epidemiology, public health, environmental sciences, ecology, tropical medicine, microbiology and infectious disease prevention and control were incorporated into the report.

To varying degrees we (AJM, CDB and BB) were all centrally involved in the creation of this report and therefore our summary is not that of a neutral observer. Nevertheless we take this opportunity to write from the ‘inside’ on what we see as the strengths and limitations of this document.

## Main text

First, it was a challenge to develop the report to this point, and considerable debate (and rewriting) among think-tank members has occurred. At the heart of this report is a growing concern about the rapid and unprecedented changes occurring in Earth’s environment and the effects such changes have on human and animal health, and social and ecological sustainability. Accumulating evidence is telling us that eco-social drivers, including climate change, energy insecurity, deforestation, antimicrobial and insecticide resistance, urban expansion, land clearance, agricultural intensification, habitat degradation, urban-industrial air pollution and counterfeit drugs are harmful to human health and the environment. Their combined influence is often synergistic; that is greater than their simple sum. These drivers already harm food yields and water availability in many regions, as well as influence the biology of microbes and the epidemiology of infectious diseases. Not surprisingly, the world’s most vulnerable, i.e., people living in poverty, older people, women and children, pay the highest price in disease and disability, compromised immunity and poor health outcomes as a result of these changes.

The report’s intent is neither to catalogue a litany of ills nor to duplicate what is already well-known (perhaps in some parts of the report we have done so – though what is well-known to environmentalists may be under-recognized by health workers). Exploring interconnections between eco-social drivers and their relationship to infectious diseases requires moving into unfamiliar analytic territory, using an approach that can dilute the specificity of more conventional information about particular diseases and their main cause(s). Indeed, there is some risk – at least in the early stages – that such an approach will meet resistance from the policy and research realms because of the perceived complexity of these problems when analysed within a systems context.

A further risk is that this integrative approach, with its attention to social, commercial, industrial and environmental practices, represents advocacy on behalf of people with limited political and economic power. Drawing attention to the contributory role of the various upstream drivers challenges powerful interests, such as companies involved in large-scale agro-industry or the mining and combustion of fossil fuels.

The report gathers historical and contemporary examples from across the world to catalogue the human and ecological harm from inaction and/or ill-conceived development as well as showcase ‘good news stories’ and the possibilities of well-thought out development and clever thinking. Noteworthy examples from the report include: biofuel plantations in Columbia, which may accelerate the spread of Chagas disease in South America; climate change and the potential for the northward spread of *Schistosoma japonicum* in China; and the emergence of infectious diseases such as SARS likely to result from the combination of intensive farming of civet cats and raccoon dogs, increased human-animal interactions and global travel.

The combined impact of these large-scale, often systemic, environmental changes may be a decline in the quality of human civilization this century, affecting critical elements needed for health such as food production and delivery systems, disaster relief, adequate and safe water supply and other core elements of public health. Conversely, the benefit to human population health from avoidance of such dismal scenarios would be great. The probability of that beneficial outcome can be maximized by a shift to sustainable technologies, practices and social priorities, including keeping within the global “carbon budget” and increasing our efforts in areas proven to accelerate the reduction of poverty, such as educating girls.

Avoiding a harmful destiny may result in future generations not fully understanding the importance of these health-promoting actions. Perhaps an analogy exists in the comparative reduction in tension about nuclear war. Today, most people under the age of 30 do not share the same understanding of the threat of nuclear war compared to older people, in part because they have not lived for several decades with this threat as a real possibility. However, few would argue that young people should face the equivalent level of threat to fully appreciate the benefits of relative global peace and non-nuclear arms proliferation. Similarly, reducing the risk of overwhelmingly adverse global environmental change would be a wonderful gift to the next generation, even if most will not fully understand the significance of this gift. Unfortunately, to date, the world is far from bestowing that sustainable legacy.

## Discussion

There are two main messages that our report seeks to convey to researchers, policy-makers and funders. First, there are complex inter-linkages, often interactions, between different, pervasive, ‘upstream’ influences on infectious diseases of poverty. Second, inter-disciplinarity and inter-sectoral thinking are critical to future research to solve some of these problems. The real world in which we live comprises complex eco-social systems, and the optimal points and modes of intervention, to reduce infectious disease risks, are best identified within that non-reductionist real-world context.

A systems-based approach is advocated in the report accompanied by numerous examples of such an approach. The constructs and methods are described comprehensively, representing the fruits of a several year-long process of discussion and deliberation. Sufficient details are included for the reader to better understand how basic science, environmental and socio-demographic changes and conditions, population health and health systems research were integrated within a systems frame.

The report also describes how to apply the inter-disciplinary ideas and methods used in the report’s preparation to assess research priorities. Multiple criteria to assess each priority were developed (for example, value for money, feasibility and impact on reduction of disease burden). These criteria helped identify not only the key research priorities but also helped distinguish between research topics that might offer immediate benefits vis-à-vis research that requires longer gestation before the benefits can be seen.

This ‘thematic’ report emphasises not only the urgent need for action but, more importantly, it makes clear how research that is appropriately integrated across relevant disciplines or sectors can contribute to actually reducing the disease burden; where such research might be targeted; and what epistemological thinking should inform such work. This important step moves beyond the rhetoric of stated principles and goals to strategizing about actual implementation and planning. By summarizing the short, medium and long term strategic thinking and planning necessary to effect such change, valuable practical insights are offered to researchers, policy-makers and funders to assist their work.

This pragmatic approach also helps the report side-step the politics that have dogged the climate change debate. The report displays a clear awareness about the tenor of these debates and recognises the need to avoid falling into the ‘climate science trap’ to the exclusion of other eco-social drivers. By careful attention to the science and to language, the authors have minimised the likelihood of this report being appropriated by those with fervent political agendas. Circumspection in some areas and decisive writing in others give balance to this publication.

## Conclusion

Hopefully, what has emerged is a road-map that identifies areas where future study should occur, and an explanation of how addressing these research priorities will reduce the burden of disease and the harmful effects of eco-social change on the wellbeing, health and survival of humans and other species. Such an approach should be of benefit to both humankind and the environment.

### Read the report

WHO/TDR: *Research priorities for the environment, agriculture and infectious diseases of poverty: Technical report of the TDR Thematic Reference Group on Environment, Agriculture and Infectious Diseases of Poverty*. Geneva: WHO; 2013. Online copy available from http://who.int/tdr/publications/environment/en/.

### Top 10 research priorities put forward in the report

1. Develop integrated preventive public health strategies for infectious diseases of poverty.

2. Develop and test novel intersectoral control of neglected tropical diseases.

3. Influence funding agencies to support inter-disciplinary approaches to infectious diseases of poverty.

4. Determine how to link health, veterinary and wildlife surveillance systems.

5. Determine which population groups are most vulnerable to climate change.

6. Determine the interactions between agriculture, water use and infectious diseases of poverty.

7. Apply systems-based research to environmentally induced transmission pathways of vector-borne diseases.

8. Assess the impacts of novel approaches such as community-led total sanitation on helminth infections.

9. Assess the impacts of water management projects on disease.

10. Develop and assess community-based vector-borne disease control models.

## Competing interests

AJM was the Chair of the WHO/TDR Thematic Reference Group on Environment, Agriculture and Infectious Diseases of Poverty (TRG 4). CDB was a reference group member and main editor of the TRG4 report. BB provided research assistance in the report’s preparation.

## Authors’ contributions

BB prepared the first draft of this commentary. CDB and AJM made substantive and significant comments and contributed to the final manuscript. All authors read and approved the final manuscript.

## Supplementary Material

Additional file 1 Multilingual abstracts in the six official working languages of the United Nations. Click here for file

