# Supplementary material for: In an interconnected world: joint research priorities for the environment, agriculture and infectious disease
Source: Infect Dis Poverty. 2014 Jan 28;3:2. doi: 10.1186/2049-9957-3-2 (PMC3906909; doi:10.1186/2049-9957-3-2)
Supplement: Additional file 1 — Multilingual abstracts in the six official working languages of the United Nations. [file 2049-9957-3-2-S1.pdf]

Translation of the abstract into the six official working languages of the United Nations

في هذا العالم المترابط: بحوث مشتركة ذات أولوية في مجال البيئة والزراعة والأمراض المعدية

Bianca Brijnath, Colin D. Butler and Anthony J. McMichael

ملخص

في عام 2008 قام برنامج الأمم المتحدة الإنمائي / برنامج اليونيسف / البنك الدولي / منظمة الصحة العالمية, الخاص بالبحوث والتدريب في مجال أمراض المناطق الاستوائية , بتكليف عشرة مراكز أبحاث للعمل على تحديد الامراض والجماعات المرجعية الموضوعية لتحديد الأولويات البحثية العليا, التي من شأنها دفع جدول أعمال البحوث حول الأمراض المعدية الناتجة عن الفقر، وبالتالي المساهمة في تحسين صحة الإنسان. وقد صدر أول هذه التقارير الموضوعية للمجموعة المرجعية - عن البيئة والزراعة والأمراض المعدية الناجمة عن الفقر - مؤخرا. في هذه المقالة نستعرض، من وجهة نظر متعمقة ، نقاط القوة والضعف في هذا التقرير الموضوعي للمجموعة المرجعية وتسليط الضوء على الرسائل الرئيسية لصانعي السياسات والممولين والباحثين.

Translated from English version into Arabic by Hany Adel, through

TRANSLATORS WITHOUT BORDERS

纵横交错世界中：环境、农业和传染病的优先联合研究领域

Bianca Brijnath, Colin D. Butler and Anthony J. McMichael

摘要

联合国儿童基金会/联合国开发计划署/世界银行/世界卫生组织热带病科研和培训特别规划署（TDR）在2008年委任了十个智囊团分别以疾病专项组和专题咨询组开展工作，旨在确定能推动贫困所致传染病研究的优先研究领域，为改善人类健康做贡献。第一份关于环境、农业和贫困引起的传染病专题咨询组研究报告近期已发布。本文我们以圈内人的角度综述了这份报告的优缺点，为政策制定者、资助者以及研究人员提供重要的信息。

Translated from English version into Chinese by YIN Jian-hai, through

CDC CHINESE CENTER FOR DISEASE CONTROL AND PREVENTION
NATIONAL INSTITUTE FOR PARASITIC DISEASES

Dans un monde interconnecté : domaine prioritaire commun de recherche pour l'environnement, l'agriculture et les maladies infectieuses

Bianca Brijnath, Colin D. Butler et Anthony J. McMichael

Résumé

En 2008, sous l'égide de l'UNICEF, l'UNDP, la Banque mondiale et l'OMS, le Programme spécial de recherche et de formation concernant les maladies tropicales (TDR) a mandaté dix cellules de réflexion pour travailler sur des groupes de références thématiques et spécifiques de maladies afin d'identifier les grands axes qui ferait avancer le programme de recherche en matière de maladies infectieuses liées à la pauvreté, et contribuer de la sorte à l'amélioration de la santé humaine. Le premier des rapports des groupes de référence thématiques – sur l'environnement, l'agriculture et les maladies infectieuses liées à la pauvreté – a été récemment publié. Dans le présent article, nous abordons, d'un point de vue interne, les atouts et faiblesses de ce rapport et mettons en exergue les messages importants destinés aux décideurs, bailleurs de fonds et chercheurs,

Translated from English version into French by Leroym, through

TRANSLATORS
WITHOUT BORDERS

Взаимосвязанный мир. Приоритеты совместных исследований для экологии, сельского хозяйства и лечения инфекционных заболеваний

Бьянка Бриджнат, Колин Д. Батлер и Энтони Дж. Макмайкл

Резюме

В 2008 году Особая научно-исследовательская и образовательная программа по изучению тропических заболеваний, проводимая совместно ЮНИСЕФ, Программой развития ООН, Всемирным банком и ВОЗ, объединила вокруг себя десять видных ученых, которым предстояло выявить основные исследовательские приоритеты по группам заболеваний и другим тематическим группам для расширения текущих исследовательских задач в отношении инфекционных заболеваний, вызванных бедностью, и повышения общего состояния здоровья населения нашей планеты. Недавно были опубликованы доклады по первой тематической группе, включающей экологию, сельское хозяйство и инфекционные заболевания, вызванные бедностью. В статье представлен внутренний анализ сильных и слабых сторон данного тематического отчета с расстановкой необходимых акцентов для политиков, спонсоров и исследователей.

Translated from English version into Russian by Irina Zayonchkovskaya, through

En un mundo interconectado: prioridades de investigación conjunta en medio ambiente, agricultura y enfermedades infecciosas

Bianca Brijnath, Colin D. Butler y Anthony J. McMichael

Resumen

En 2008, el Programa Especial de UNICEF/PNUD/Banco Mundial/OMS de investigaciones y enseñanzas sobre enfermedades tropicales (TDR en sus siglas en inglés) encargó a diez organizaciones que trabajaran en grupos de referencia temáticos y sobre enfermedades concretas con el objetivo de identificar las prioridades para mejorar el programa de investigación de las enfermedades infecciosas de la pobreza, contribuyendo de esta forma a mejorar la salud de la humanidad. El primero de los informes de los grupos de referencia temáticos –sobre medio ambiente, agricultura y las enfermedades infecciosas de la pobreza–, fue publicado recientemente. En este artículo analizamos, desde una perspectiva interna, las fortalezas y debilidades de este informe y señalamos los mensajes clave para los diseñadores de políticas públicas, financiadores e investigadores.

Translated from English version into Spanish by Teresa Alvarez, through

TRANSLATORS
WITHOUT BORDERS
